# Triple-dose bolus versus continuous infusion of tranexamic acid: impacts on clinical outcomes in isolated coronary artery bypass surgery

**DOI:** 10.1186/s13019-026-03877-5

**Published:** 2026-02-12

**Authors:** Osman Uzundere, Mahmut Yargı, Selen Topalel, Ahmet Serkan Aydın, Meral Erdal Erbatur, Hülya Tosun Söner, Reşit Saruhan, Cem Kıvılcım Kaçar, Erhan Gökçek

**Affiliations:** 1 Department of Anesthesiology and Reanimation, TR HSU Diyarbakır Gazi Yaşargil TRH, 21100, Diyarbakır, Turkey; 2 Department of Cardiovascular Surgery, TR HSU Diyarbakır Gazi Yaşargil TRH, Diyarbakır, Turkey; 3 Department of Anesthesiology and Reanimation, Dicle University Medical Faculty, Diyarbakır, Turkey

**Keywords:** Coronary artery bypass grafting, Tranexamic acid dosing strategies, Triple-bolus regimen, Postoperative bleeding, Cardiopulmonary bypass

## Abstract

**Background:**

In cardiac surgery, intraoperative tranexamic acid (TXA) is commonly used and highly recommended approach for managing perioperative bleeding. Nevertheless, a standardized dose and administration protocol remains undefined. This study compares the efficacy of intraoperative triple-dose intravenous (IV) bolus TXA versus IV bolus followed by continuous IV infusion in preventing postoperative bleeding in patients undergoing isolated coronary artery bypass grafting (CABG).

**Methods:**

The study included 93 patients who underwent elective isolated CABG between August 14, 2023, and October 14, 2024. Patients received either triple-dose IV bolus TXA (Group 1, *n* = 53) or IV bolus followed by continuous IV infusion (Group 2, *n* = 40) during surgery.

**Results:**

Postoperative bleeding was lower in Group 1 at all assessed time points (1 h: 116 vs. 146 mL; 6 h: 253 vs. 332 mL; 24 h: 589 vs. 713 mL). However, a statistically significant difference was found only at 6 h (*p* = 0.03), representing an approximately 24% relative reduction in bleeding volume compared with Group 2. No significant differences were observed in transfusion requirements (packed red blood cells *p* = 0.85; fresh frozen plasma *p* = 0.55). Three patients in Group 2 required reoperation due to bleeding (*p* = 0.07), and one late mortality occurred in Group 2 (*p* = 0.43).

**Conclusion:**

This study suggests that triple-dose IV bolus TXA administration may be both an effective and safe strategy for preventing postoperative bleeding in isolated CABG surgery.

**Trial registration:**

The study was registered at the Clinical Trial Registry (NCT05994989, August 8, 2023) on.

**Supplementary Information:**

The online version contains supplementary material available at 10.1186/s13019-026-03877-5.

## Introduction

Coronary artery bypass grafting (CABG) is among the most commonly performed cardiac surgeries worldwide, offering significant improvements in both survival rates and quality of life for selected patients. Despite approximately 200,000 isolated CABG surgeries being performed annually in the United States, the number of CABG procedures has nearly halved since the early 2000 s due to the increased use of percutaneous coronary interventions and other minimally invasive cardiac procedures [1, 2]. Although CABG is a frequently performed surgical procedure, it carries numerous risks, among which intraoperative and postoperative coagulopathy are particularly significant. Coagulopathy in CABG may arise from several factors, such as the need for high-dose anticoagulation, the use of cardiopulmonary bypass (CPB), and hemodilution. These factors collectively contribute to platelet destruction and thrombin generation, ultimately enhancing fibrinolytic activity [3, 4]. In these patients, both the high requirement for blood product transfusions (50–60%) and reoperation due to bleeding are associated with adverse clinical outcomes [5]. Consequently, perioperative bleeding management remains a pertinent issue in cardiothoracic surgery.

Guidelines issued by the European Association for Cardio-Thoracic Surgery (EACTS) and the European Association of Cardiothoracic Anaesthesiology (EACTA), including the recently updated 2024 recommendations, offer comprehensive guidance on the management of perioperative bleeding in cardiac surgery patients [6, 7]. These guidelines highlight the widespread use of antifibrinolytic agents to reduce bleeding, transfusion requirements, and the risk of reoperation due to bleeding [6, 7]. Tranexamic acid (TXA), a lysine analog, is one of the most frequently used antifibrinolytic agents in surgical procedures with a high risk of bleeding, including cardiac surgeries, due to its hemostatic efficacy and limited side effects. TXA exerts its effect by blocking the binding of plasminogen to fibrin, thereby stabilizing the blood clot [8, 9]. The most significant reported side effect of TXA is convulsive seizures, particularly at high doses [6, 7]. Recent research has primarily focused on determining the minimum effective dose of TXA to mitigate this risk. Studies have demonstrated that earlier high-dose regimens, such as 100 mg/kg, have been replaced with significantly lower doses that are equally effective [3, 10–14].

In clinical practice, TXA administration protocols in cardiac surgery vary between centers. While some centers utilize one or more intravenous (IV) bolus doses, others apply an IV bolus followed by a continuous IV infusion [3, 15]. Although the efficacy of TXA in reducing perioperative bleeding in cardiac surgery is well-established, direct comparative evidence between these two commonly used strategies in adult CABG remains scarce. Most existing studies favor bolus-plus-infusion regimens, whereas data supporting multiple-bolus dosing are predominantly based on pediatric cardiac surgery populations [10, 16]. To date, no prospective clinical study has evaluated the clinical impact of a triple-dose IV bolus regimen in adult CABG patients. Therefore, there is a critical need to determine whether a multiple-bolus strategy can provide comparable—or potentially superior—hemostatic effectiveness while minimizing prolonged TXA exposure.

This study aims to compare the efficacy of triple-dose IV bolus versus a single IV bolus followed by continuous infusion in adult patients undergoing isolated CABG, addressing a significant gap in the current clinical evidence. The primary outcome is to evaluate postoperative bleeding amounts, while the secondary outcomes include comparisons of perioperative clinical data, blood and blood product usage, postoperative complications, and lengths of stay in the intensive care unit (ICU) and hospital.

## Materials and methods

### Study design

This prospective observational study was conducted at Diyarbakır Gazi Yaşargil Education and Research Hospital between August 14, 2023, and October 14, 2024. The necessary permission for the study was obtained from the ethics committee of SBÜ Diyarbakır Gazi Yaşargil Training and Research Hospital (approval no. 408, May 26, 2023). The trial was registered with ClinicalTrials.gov (NCT05994989, August 8, 2023). The study adhered to the principles of the 2013 Declaration of Helsinki.

### Inclusion and exclusion criteria

Patients aged 18 years or older who underwent elective isolated CABG and received either the triple-dose IV bolus or the IV bolus followed by continuous IV infusion TXA protocol were included. Patients were excluded if they underwent procedures other than CABG, CABG combined with valve surgery (aortic or mitral), redo CABG or other cardiac repairs, or received TXA using a regimen different from the two predefined protocols. Patients who died within the first 24 postoperative hours were also excluded. This criterion was implemented to ensure that TXA efficacy could be evaluated over a complete 24-hour postoperative bleeding assessment period, as early (< 24 h) mortality in CABG is typically related to major surgical complications or emergent reoperations rather than TXA exposure. Additional exclusion criteria included age < 18 years, emergency surgery, preoperative liver or renal failure, cerebrovascular events, bleeding diathesis, a history of seizures, or legal/mental incapacities.

### Perioperative procedures

All patients were preoperatively evaluated by an anesthesiologist, and written informed consent was obtained. Preoperative antiplatelet management followed a standardized institutional protocol: clopidogrel was discontinued 5 days and ticagrelor 3 days prior to surgery, while low-dose aspirin was continued until the morning of the operation. Standard intraoperative monitoring included electrocardiography, pulse oximetry, non-invasive blood pressure measurement, and hemodynamic monitoring. Anesthesia induction and maintenance were performed per institutional protocols, and all surgeries were conducted by two experienced cardiovascular surgeons. Hemodynamic management followed a goal-directed approach, with restrictive fluid administration (baseline 2 mL/kg/h) and the use of vasoactive agents in cases of hypotension rather than additional crystalloid or colloid infusion. No colloids were used intraoperatively, and stroke volume variation (SVV), pulse pressure variation (PPV), cardiac index (CI), and systemic vascular resistance (SVR) were monitored using the MostCare system. Patients were transferred intubated to the cardiovascular intensive care unit (ICU) for postoperative management. All patients were managed postoperatively according to a standardized fast-track extubation protocol in our cardiovascular ICU. Mechanical ventilation was discontinued once predefined hemodynamic, respiratory, and metabolic stability criteria were met. As this protocol was uniformly applied throughout the study period, extubation practices did not differ between the two groups.

A standardized institutional transfusion protocol was applied to all patients. RBC transfusion was administered when hemoglobin dropped below 8 g/dL or when signs of inadequate oxygen delivery were present (lactate elevation or mixed venous oxygen saturation < 60%). Platelet transfusion was guided by active bleeding with thrombocytopenia, and fresh frozen plasma (FFP) was administered for prolonged coagulation times in the presence of ongoing bleeding. Transfusion decisions were therefore not made at clinician discretion, minimizing bias between groups.

### Patient grouping

During the two-year study period, two different TXA dosing regimens were used sequentially at our center as part of routine institutional practice. In the initial phase, the standard protocol was the triple-dose bolus regimen described by Chauhan et al., consisting of 10 mg/kg IV administered at three intraoperative time points (after anesthesia induction, during CPB, and after protamine; total approximately 30 mg/kg) [9]. In the later phase of the study period, the department adopted a bolus-plus-infusion protocol, consisting of a 10 mg/kg IV bolus administered over 15 min after anesthesia induction, followed by a continuous IV infusion of 2 mg/kg/h for 8 h (total approximately 26 mg/kg). Although the administration protocols defined approximate total TXA exposure (~ 30 mg/kg for the triple-bolus regimen and ~ 26 mg/kg for the bolus-plus-infusion regimen), the exact patient-specific cumulative doses were not recorded in the electronic data system. This protocol change reflected an institutional practice update rather than patient-specific or clinician-directed decision-making. All patients undergoing elective isolated CABG during these periods were enrolled consecutively, and the TXA regimen each patient received was determined exclusively by the active institutional protocol at the time of surgery. No randomization, matching, stratification, or discretionary assignment was performed.

**Fig. 1:**
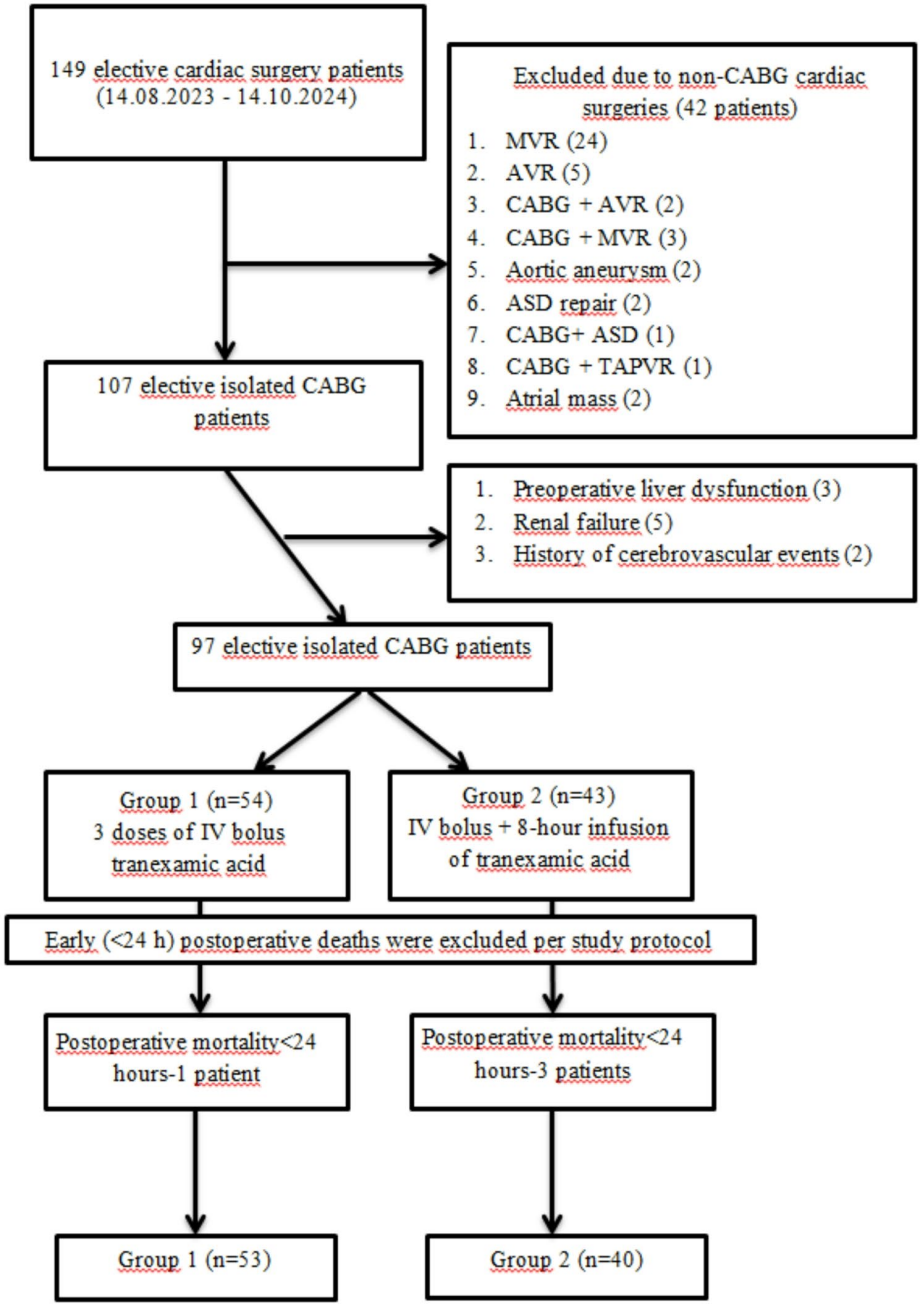
Study flow diagram showing patient inclusion and exclusion

A total of 93 patients who received one of these two regimens during the specified period were included in the study. Patients were divided into two groups based on the TXA regimen: Group 1 (*n* = 53) received the triple-dose IV bolus, while Group 2 (*n* = 40) received the IV bolus followed by continuous IV infusion. The study flowchart is presented in Fig. 1.

### Data collection

The demographic, preoperative, intraoperative, and postoperative data of the patients included in the study were systematically recorded. In cases of missing or incorrect data, patient files and the hospital information system were reviewed to ensure accuracy. The recorded data included patient age, sex, body surface area (BSA), presence of comorbidities, smoking history, number of vessels to be operated on, ejection fraction (EF), New York Heart Association (NYHA) heart failure classification [17], European System for Cardiac Operative Risk Evaluation (EuroSCORE) II [18], and preoperative laboratory values. In this study, BSA was used instead of body mass index (BMI) as a more relevant parameter in cardiac surgery research. Additionally, the intraoperative protocol for TXA administration was recorded. Other intraoperative data included activated clotting time (ACT) values (measured before CPB, during CPB, and after protamine), cross-clamp (CC) and CPB durations, surgery and anesthesia durations, intraoperative urine output, intraoperative inotropic requirements and types of inotropes used, and intraoperative quantities of packed red blood cells (PRBC) and fresh frozen plasma (FFP) transfused were documented.

Postoperative data encompassed bleeding amounts from thoracic and mediastinal drainage tubes at postoperative hours 1, 6, and 24. These measurements were recorded as cumulative drainage volumes without hourly resets. It also included postoperative PRBC, FFP, platelets usage, laboratory values, ICU and hospital stay durations, ICU complications, and mortality status.

### Primary outcome

The primary outcome was the amount of bleeding from thoracic and mediastinal drainage tubes at postoperative hours 1, 6, and 24.

### Secondary outcome

Intraoperative and postoperative clinical data, transfusion requirements, postoperative complications, and ICU/hospital length of stay.

### Statistical analysis

The sample size was determined using G-Power software (version 3.1.9.4; University of Kiel, Kiel, Germany) based on 24-hour bleeding data from a previous study (10). A minimum of 40 patients per group was calculated to be required (assuming a two-tailed alpha error of 0.05, a power of 0.80, an allocation ratio of N2/N1 = 1, and an effect size of 0.63). At the time of study planning, no adult CABG studies directly compared triple-bolus and bolus-plus-infusion TXA regimens or reported extractable mean ± SD bleeding values suitable for a formal power calculation. Therefore, the dataset from Chauhan et al. (pediatric cardiac surgery), which provided clearly defined postoperative bleeding means and variability measures aligned with our primary endpoint, represented the closest available quantitative reference.

Statistical analyses were performed using SPSS 16.0 software for Windows (SPSS Inc., Chicago, IL, USA). Categorical variables were analyzed using Chi-square and Fisher’s exact tests and presented as frequencies and percentages. The Kolmogorov-Smirnov test was used to assess whether continuous data were normally distributed. Normally distributed continuous variables were analyzed using Student’s t-test and presented as mean ± standard deviation. Non-normally distributed data were analyzed using the Mann-Whitney U test and presented as median (interquartile range). A p-value of < 0.05 was considered statistically significant for all comparisons.

## Results

A total of 93 patients were included in the study. The mean age of the patients was 60.8 ± 8.8 years, and 74% were male. The most common comorbidities were hypertension (53.8%) and diabetes mellitus (48.4%). When the demographic, clinical, and preoperative characteristics of the patients were compared, no significant differences were found between the two groups (*p* > 0.05). Details are provided in Table 1, which summarizes the demographic, clinical, and preoperative characteristics of the patients in both groups.

**Table 1:** Demographic and preoperative characteristics of patients

Characteristic	Group 1	Group 2	*p*
	(*n* = 53)	(*n* = 40)	
Age (Year)^*^	60,75 ± 8,7	60,85 ± 9,1	0.95
Sex F/M (n)	13/40	11/29	0.74
BSA (m2) ^&^	1,82 (1,73 − 1,96)	1,82 (1,75 − 1,98)	0.73
Comorbidity, n (%)	46(58,2)	33(41,8)	0.56
Diabetes	22(48,9)	23(51,1)	0.12
Hypertension	27(54)	23(46)	0.53
COPD	10(62,5)	6(37,5)	0.62
Smoking, n (%)	12(60)	8(60)	0.75
Ejection fraction (%)^&^	55(45–60)	55(50–60)	0.67
Euroscore II^&^	1 (0,8 − 1,3)	0,98 (0,71 − 1,41)	0.70
NYHA class 2/3 (n)	35/18	31/9	0.37
Hemoglobin (g/dl) ^&^	14 (13–15)	14,4 (13–15)	0.41
Hematocrit (%)*	42 ± 4,5	42,9 ± 4,9	0.36
Platelet (× 103/uL)*	266,3 ± 74,4	247 ± 56,2	0.17
Prothrombin time (s) ^&^	12 (11,6–12,7)	11,9 (11,2–12,6)	0.41
Activated partial thromboplastin time (s)*	32,8 ± 4,5	34,3 ± 6,4	0.17
Blood urea nitrogen (mg/dL) ^&^	39 (28,5–44)	33,5 (29–44,5)	0.75
Creatinine (mg/dL) ^&^	0,8 (0,7 − 0,9)	0,8 (0,7 − 1,03)	0.15
Alanine aminotransferase (U/L) ^&^	20 (13–26,5)	18,5 (16–37,5)	0.15
Aspartate aminotransferase (U/L) ^&^	20 (17–28,5)	23,5 (18–50,2)	0.07

^*^Mean±SD; ^&^Median (interquartile range)

BSA: Body surface area; COPD: Chronic obstructive pulmonary disease; NYHA: New York Heart Association

The majority of patients undergoing CABG surgery required intervention on three or more vessels (65.6%). No significant differences were observed between the two groups regarding intraoperative characteristics, including ACT values, cross-clamp time, CPB duration, total surgery time, and anesthesia duration (all *p* > 0.05). Similarly, intraoperative transfusion of PRBCs and FFP did not differ significantly between the groups (*p* = 0.57 and *p* = 0.67, respectively). Intraoperative inotrope use was also comparable, with noradrenaline administered in 64.5% of patients, dopamine in 83.9%, and dobutamine in 12.9%, without significant between-group differences. Detailed intraoperative characteristics are presented in Table 2.

**Table 2:** Intraoperative characteristics of patients

Characteristic	Group 1	Group 2	*p*
	(*n* = 53)	(*n* = 40)	
CABG, n (%)			0.32
≤ 2 vessel	16(30,2)	16(40)	
≥ 3 vessel	37(69,8)	24(60)	
ACT before CPB (s)^*^	137,1 ± 30,8	137,2 ± 25,5	0.98
ACT during CPB (s) ^&^	531 (463,5–612,5)	520,5 (429,7–692)	0.92
ACT after protamine (s) ^&^	117 (107–128)	120,5 (111,5–131,5)	0.26
CC (min)^*^	59,5 ± 23,5	64,1 ± 32,2	0.42
CPB (min)^*^	95,1 ± 29,3	100,3 ± 41,3	0.48
Duration of surgery (min)^*^	215,6 ± 46,3	212,9 ± 54,7	0.80
Duration of anesthesia (min) ^&^	240(205–260)	240(198–270)	0.57
Urine output (ml) ^&^	700 (500–1200)	930 (527–1300)	0.52
Inotrope, n (%)	47(58)	34(42)	0.60
Noradrenaline	35(58,3)	25(41,7)	0.72
Dopamine	46(59)	32(41)	0.37
Dobutamine	7(58.3)	5(41.7)	0.92
PRBC (unit) ^&^	0(0–1)	0(0–1)	0.57
FFP (unit) ^&^	2(0–2)	2(0–2)	0.67

*Mean±SD; ^&^Median (interquartile range)

CABG: Coronary artery bypass graft; ACT: Activated clootting time; CPB: Cardiopulmonary bypass; CC: Cross-clamp; PRBC: Packed red blood cells; FFP: Fresh frozen plasma

**Fig. 2:**
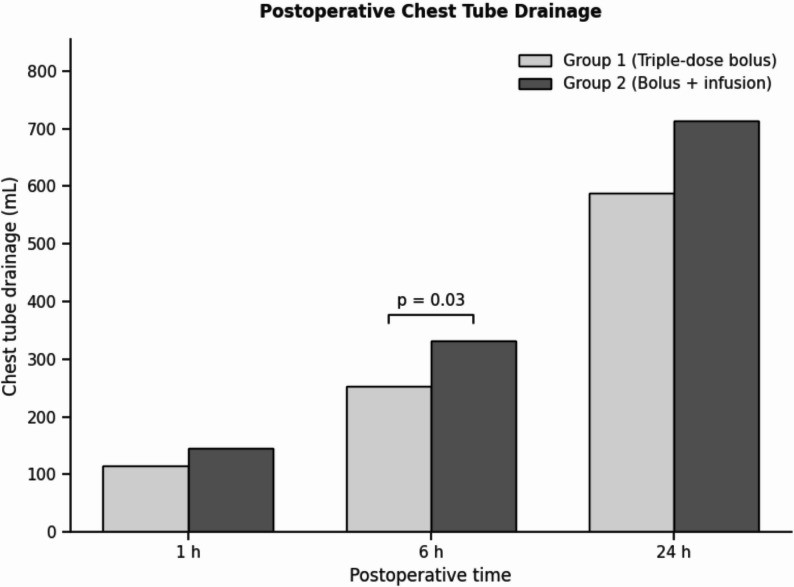
Postoperative chest tube drainage at 1, 6, and 24 h

**Fig. 3:**
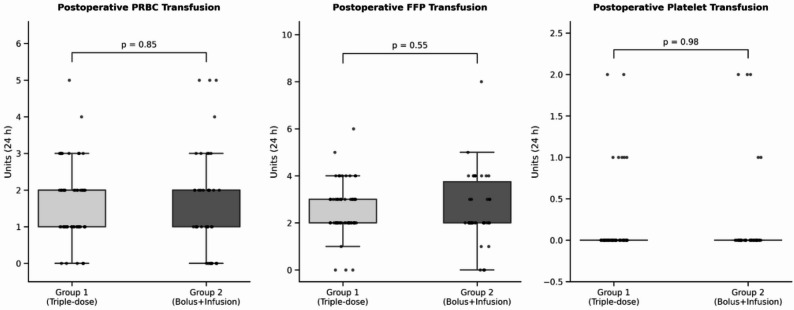
Postoperative transfusion of blood products within the first 24 h. PRBC: Packed Red Blood Cells; FFP: Fresh Frozen Plasma

When comparing the groups in terms of the primary outcome, which was postoperative thoracic and mediastinal drainage at 1, 6, and 24 h, Group 1 exhibited lower bleeding volumes than Group 2 at all time points (116 mL vs. 146 mL at 1 h; 253 mL vs. 332 mL at 6 h; and 589 mL vs. 713 mL at 24 h). The relative difference was most pronounced at 6 h, with approximately 24% lower bleeding in Group 1. However, this difference reached statistical significance only at the 6-hour time point (*p* = 0.03), while the reductions at 1 h (≈ 20%) and 24 h (≈ 17%) did not achieve statistical significance (*p* = 0.17 and *p* = 0.06, respectively) (Fig. 2). No significant difference was observed between the groups in terms of postoperative PRBCs, FFPs, and platelets within the first 24 h (p values: 0.85; 0.55; 0.98) (Fig. 3).

During the postoperative period, respiratory failure was identified as the most common complication (10 patients, 10.8%), followed by hypotension (8 patients, 8.6%) and arrhythmias (8 patients, 8.6%). Regarding safety outcomes, no postoperative seizures or thromboembolic events were observed in either group (Table 3). There were no statistically significant differences in the incidence of postoperative complications, including respiratory failure, hypotension, and arrhythmias, between the two groups. There were also no significant differences in mortality, ICU stay duration, or hospital stay duration between the groups (p values: 0.43; 0.22; 0.26). Importantly, three patients in Group 2 required reoperation due to bleeding, and one mortality occurred after 24 h in this group. All reoperations for bleeding in Group 2 (*n* = 3) were attributed to surgically correctable causes, such as anastomotic site oozing or generalized mediastinal bleeding. No cases were associated with coagulopathy, graft failure, or thrombotic complications. One postoperative death included in the final analysis occurred in Group 2 on postoperative day 9 due to acute respiratory distress syndrome (ARDS). Early (< 24 h) deaths (Group 1: *n* = 1, Group 2: *n* = 3) were excluded per predefined study criteria. Details are provided in Table 3.

**Table 3:** Postoperative outcomes and complications

Characteristic	Group 1	Group 2	*p*
	(*n* = 53)	(*n* = 40)	
ICU complication, n (%)	18(48,6)	19(51,4)	0.18
Hypotension	4(4,3)	4(4,3)	0.72
Arrhythmia	3(3,2)	5(5,4)	0.28
Respiratory failure	5(5,4)	5(5,4)	0.63
Bleeding requiring reoperation	0(0)	3(3,2)	0.07
Postoperative seizures, n (%)	0(0)	0(0)	
Thromboembolic events, n (%)	0(0)	0(0)	
Mortality, n (%)	0(0)	1(100)	0.43
Length of stay in the ICU (day) ^&^	2(2–3)	3(2–4)	0.22
Length of stay in the hospital (day)^&^	10(8–14)	9(7–12)	0.26

^&^Median (interquartile range)

ICU: Intensive care unit

## Discussion

This study compared two different intraoperative TXA administration methods in elective isolated CABG surgery. The findings demonstrated that patients receiving intraoperative triple-dose IV bolus TXA had significantly lower postoperative bleeding volumes and fewer cases requiring reoperation due to bleeding compared to patients receiving a single IV bolus followed by an 8-hour infusion.

Recent guidelines on blood management in cardiac surgery from various countries strongly recommend the prophylactic use of TXA to reduce blood loss, transfusion requirements, and reoperation risks [3, 5, 7, 19–21]. However, there is still no consensus on the optimal TXA dosage and administration strategy for cardiac surgery. The 2021 meta-analysis by Zufferey et al., including 64 randomized controlled trials and 18 observational studies, identified 73 different TXA regimens, highlighting the significant variability in dosing strategies [12]. Similarly, a 2017 survey by Spence et al. showed that 68.4% of cardiac anesthetists in Canada preferred a bolus followed by infusion, whereas only 12% used multiple IV bolus doses [15]. These findings reflect the lack of standardization in TXA administration and underscore the need for further comparative studies. The study design was grounded in the 2017 EACTS/EACTA Guidelines [6], though we also acknowledge the recently updated 2024 EACTS guidelines [7], which continue to support TXA use while emphasizing the need for personalized bleeding management strategies.

The sequential use of two different TXA strategies during the study period reflects our institutional dosing evolution. The triple-bolus regimen was implemented during the earlier phase based on established literature describing repeated dosing at key intraoperative phases to counteract CPB-induced hemodilution. Recent pharmacodynamic evidence, including the 2024 BJA report [22], also demonstrates that the antifibrinolytic effect of a single bolus diminishes substantially in the early postoperative hours. This supports the rationale for repeated intraoperative dosing during procedures with prolonged CPB times, where fibrinolytic activity typically peaks. It is important to note that our study focused exclusively on on-pump CABG. Consequently, these findings may not apply to off-pump CABG, where the absence of cardiopulmonary bypass results in a different fibrinolytic profile and alters the pharmacokinetic requirement for intra-procedural TXA redosing.

One of the key concerns regarding TXA administration is the dose-response relationship and its impact on postoperative seizure risk. Many studies have focused on determining the minimum effective dose to balance hemostatic benefits with potential neurological complications [8, 10, 23]. While bolus + infusion regimens are believed to achieve more stable plasma concentrations and reduce fibrinolytic activity over a prolonged period, some studies have reported that multiple IV boluses may provide effective fibrinolysis suppression with a potentially lower seizure risk [8, 14, 19, 23].

Hodgson et al. suggested that high-dose TXA (30 mg/kg bolus + 16 mg/kg/h infusion + 2 mg/kg priming) should be used for high-risk patients, whereas low-dose TXA (10 mg/kg bolus + 1 mg/kg/h infusion + 1 mg/kg priming) is safer for low-risk cases (11). Shi et al. conducted the OPTIMAL trial, which supported this recommendation but adjusted the low-dose infusion to 10 mg/kg bolus + 2 mg/kg/h + 1 mg/kg priming to maintain plasma concentrations ≥ 20 µg/mL throughout surgery. The authors concluded that high-dose TXA is both safer and more effective than low-dose regimens [14]. Conversely, Guo et al.‘s meta-analysis of 49 randomized controlled trials found that low-dose IV infusion (bolus injection < 50 mg/kg or 10 mg/kg + 1 mg/kg/h) reduced transfusion rates as effectively as high-dose regimens without increasing seizure risk [23]. These conflicting reports highlight the complexity of defining an “optimal” regimen.

The triple-dose IV bolus regimen offers an alternative strategy. In 2004, Chauhan et al. study in pediatric cardiac surgery introduced this regimen and demonstrated its efficacy in reducing blood loss [9]. Madathil et al. later confirmed that even higher triple-bolus doses (25 mg/kg) were associated with lower blood loss without significant side effects [16]. In our study, the triple-dose IV bolus regimen resulted in consistently lower postoperative bleeding volumes, although statistical significance was observed only at the 6-hour time point.

The temporal significance observed exclusively at the 6-hour mark aligns with established pharmacokinetic and pharmacodynamic characteristics of TXA and the fibrinolytic profile following CPB [12, 24]. Fibrinolytic activation is known to peak in the early postoperative hours due to the release of tissue plasminogen activator (tPA), hemodilution, and surgical trauma [25]. Reinforcement of TXA plasma levels at key points of fibrinolytic activity in the triple-bolus regimen likely enhanced antifibrinolytic efficacy during this period. In contrast, plasma concentrations gradually decline following discontinuation of infusion in the bolus-plus-infusion protocol, resulting in comparable antifibrinolytic effects between groups by 24 h [12, 24]. Approximately 30%, 55%, and almost 90% of administered TXA is excreted at 1, 3, and 24 h, respectively [26]. Thus, the attenuation of efficacy over time is pharmacokinetically expected for both strategies and emphasizes the need for administration schedules synchronized with postoperative fibrinolytic dynamics.

Although the absolute difference in bleeding was modest and did not translate into statistically significant reductions in transfusion requirements or ICU/hospital length of stay, the clinical implications warrant attention. Numerically lower rates of reoperation due to bleeding and shorter ICU stays in the triple-bolus group may indicate meaningful benefits. Such trends could suggest improved early hemostatic stability. However, our study was not powered to detect significance in these secondary outcomes; therefore, larger randomized trials are required to confirm whether these trends translate into clinically relevant improvements.

Beyond hemostatic efficacy, the triple-bolus regimen offers practical advantages. It simplifies anesthetic management by removing the need for a dedicated infusion pump and rate calculations, thereby reducing equipment costs and the potential for programming errors, while ensuring peak plasma levels coincide with critical phases of fibrinolytic activation.

Two major concerns associated with TXA use in CABG patients are thrombotic complications and postoperative seizures. The ATACAS trial by Myles et al., which included 4,631 cardiac surgery patients, demonstrated that TXA significantly reduced blood loss, transfusion requirements, and reoperation rates without increasing thrombotic complications or 30-day mortality [19]. However, the same study noted a higher seizure incidence with high-dose TXA compared to placebo, a finding also supported by Zufferey et al.‘s meta-analysis [12]. Additionally, because TXA is renally excreted, impaired renal function may elevate plasma concentrations and increase seizure risk, underscoring the importance of dose consideration in patients with reduced renal clearance. In our study, no thromboembolic events or postoperative seizures were observed, suggesting that both regimens can be safely used in isolated CABG. This suggests that both regimens kept plasma concentrations within a safe therapeutic window, avoiding the toxicity associated with excessive accumulation.

### Limitations

Our study has several limitations. First, exact patient-specific cumulative TXA doses were not recorded in the electronic data system, although predefined dosing protocols were followed. Similarly, although intraoperative fluid management followed a standardized restrictive protocol, exact crystalloid infusion volumes were not captured in the dataset. Additionally, the total TXA dose differed slightly between the two regimens (~ 30 mg/kg vs. ~26 mg/kg), which represents a potential confounding factor. Future studies with precise dose tracking may provide deeper insight into dose–response relationships.

Second, postoperative chest tube drainage was recorded as cumulative rather than hourly reset values. Although cumulative measurements offer an overall assessment, hourly reset values might have allowed for a more detailed evaluation of temporal bleeding dynamics. Furthermore, platelet transfusion requirements were very low in both groups, limiting us from drawing meaningful comparisons for this outcome.

Finally, a further important methodological limitation is the non-randomized, time-dependent allocation of TXA regimens. The study was designed to evaluate clinical outcomes during a sequential institutional protocol change rather than as a randomized intervention. While an RCT would minimize selection bias, our observational approach allowed for the assessment of these regimens in a real-world clinical setting without deviating from the active standard of care. Since the study was performed in a single center with a limited sample size, larger multicenter studies are needed to confirm these findings.

## Conclusion

The most significant contribution of our study is that it is the first to compare triple-dose IV bolus and continuous IV infusion regimens in adult CABG patients. While previous studies suggested that continuous infusion following IV bolus may be more effective, our findings indicate that the triple-dose IV bolus regimen is an equally viable option for reducing postoperative bleeding. These results highlight the importance of TXA administration protocols in postoperative recovery and suggest that the triple-dose regimen should be considered as part of bleeding management strategies, particularly in patients where prolonged TXA exposure is a concern.

## Supplementary Information

Supplementary Material 1.

## Data Availability

The datasets used and/or analysed during the current study are available from the corresponding author on reasonable request.

## Abbreviations

ACT: Activated Clotting Time
ARDS: Acute Respiratory Distress Syndrome
BMI: Body Mass Index
BSA: Body Surface Area
CABG: Coronary Artery Bypass Grafting
CC: Cross-clamp
CI: Cardiac Index
COPD: Chronic Obstructive Pulmonary Disease
CPB: Cardiopulmonary Bypass
EACTA: European Association of Cardiothoracic Anaesthesiology
EACTS: European Association for Cardio-Thoracic Surgery
EF: Ejection Fraction
EuroSCORE: European System for Cardiac Operative Risk Evaluation
FFP: Fresh Frozen Plasma
ICU: Intensive Care Unit
IQR: Interquartile Range
IV: Intravenous
NYHA: New York Heart Association
PPV: Pulse Pressure Variation
PRBC: Packed Red Blood Cells
SCA: Society of Cardiovascular Anesthesiologists
SD: Standard Deviation
STS: Society of Thoracic Surgeons
SVV: Stroke Volume Variation
SVR: Systemic Vascular Resistance
TXA: Tranexamic Acid
