# Supplementary material for: Triple-dose bolus versus continuous infusion of tranexamic acid: impacts on clinical outcomes in isolated coronary artery bypass surgery
Source: J Cardiothorac Surg. 2026 Feb 12;21:136. doi: 10.1186/s13019-026-03877-5 (PMC12997948; doi:10.1186/s13019-026-03877-5)
Supplement: Supplementary file 1 — Supplementary Material 1. [file 13019_2026_3877_MOESM1_ESM.docx]

| **Supplementary Table 1.** Exact numerical values of postoperative chest tube drainage volumes at 1, 6, and 24 hours. | | | |
| --- | --- | --- | --- |
| **Time Point** | **Group 1 (Triple-dose bolus) (n=53)** | **Group 2 (Bolus + infusion) (n=40)** | **p-value** |
| **1st Hour (mL)** | 116,9±98,5 | 146,2±105,8 | 0.17 |
| **6th Hour (mL)** | 253,7±166,3 | 332,5±197,5 | **0.03** |
| **24th Hour (mL)** | 589.0±347,01 | 713.5±296,1 | 0.06 |
